# Hereditary angioedema caused by a premature stop codon mutation in the *SERPING1* gene

**DOI:** 10.1186/s13601-020-00360-9

**Published:** 2020-11-27

**Authors:** Ying-Yang Xu, Jian-Qing Gu, Yu-Xiang Zhi

**Affiliations:** Department of Allergy, Peking Union Medical College Hospital, Chinese Academy of Medical Sciences & Peking Union Medical College, #1 Shuaifuyuan, Wangfujing, Beijing, 100730 China

**Keywords:** C1 inhibitor, Hereditary angioedema, Mutation, Premature stop codon, *SERPING1*

## Abstract

**Background:**

Hereditary angioedema with deficient and dysfunctional C1 inhibitor (C1-INH-HAE) is a rare genetic disorder. The majority of the cases with this disease are caused by mutations in the C1-inbitor gene *SERPING1* and are classified as type 1 and type 2. We aimed to detect mutations in the *SERPING1* gene and evaluate its expression in nine probands with hereditary angioedema from nine different families.

**Methods:**

Nine probands with hereditary angioedema from nine different families and 53 healthy controls were recruited in this study. All eight exons and intron–exon boundaries in the *SERPING1* gene were amplified by PCR and then sequenced. Mutations were identified by alignment with reference sequences. mRNA expression was measured by real-time PCR.

**Results:**

All probands were diagnosed with HAE type 1. Nine mutations were found in nine patients: c.44delT, c.289C<T, c.296_303delCCATCCAA, c.538C<T, c.786_787insT, c.794 G < A, c.939delT, c.1214_1223delCCAGCCAGGA, and c.1279delC. All mutations formed a premature stop codon that might lead to the impaired synthesis of C1 inhibitor and result in the deficiency of this protein. None of the detected mutations were observed in the controls. In the C1-INH-HAE group, *SERPING1* mRNA expression was significantly reduced (20% of the normal average level) compared to controls.

**Conclusions:**

Three known and six novel mutations in the *SERPING1* gene were identified, and they produced a truncated nonfunctional C1 inhibitor without a reactive central loop. All the mutations led to reduced expression of *SERPING1* mRNA in peripheral blood and low antigenic C1 inhibitor levels.

## Background

Hereditary angioedema (HAE) is a rare disorder that presents with recurrent attacks of edema involving deep dermal/subcutaneous or mucosal/submucosal tissues [1, 2]. HAE can affect various body sites, most often the extremities, face, genitalia, airway and gastrointestinal tract [2, 3]. Upper airway obstruction and asphyxiation usually result from laryngeal edema which can be life-threatening [4, 5]. Laryngeal edema is the main cause of death in patients with HAE, the reported mortality rate ranging between 11 and 40% [5, 6].

Currently, different forms of HAE have been recognized. HAE type 1 (HAE-1) and type 2 (HAE-2) are usually autosomal dominant diseases caused by mutations in the C1 inhibitor (C1-INH) gene *SERPING1* [1, 7]*.* HAE-1 is defined by the presence of both low C1-INH levels and a functional C1-INH defect. HAE-2 is characterized by dysfunctional C1-INH with normal or elevated C1-INH (antigenic) level. Type-1 and type-2 HAE are defined as C1-INH-HAE, and are responsible for approximately 95% of HAE cases [8]. In addition, HAE with normal C1-INH (HAE nC1-INH) has been described and classified into subtypes based on genetic defects in factor XII [9], angiopoietin-1 [10], plasminogen [11], kininogen-1 heavy chain [12] or an unknown mutation.

The lack of a functional C1-INH leads to overactivation of the kallikrein-kinin system and overproduction of bradykinin. To date, approximately 748 mutations in *SERPING1* have been identified as responsible for HAE-1/2. The majority are heterogeneous, with only 10 homogeneous genetic defects reported [13]. However, only a few of them have been evaluated for the expression of *SERPING1* mRNA [14–18].

In this study, we describe the mutational findings and analyze the mRNA expression of *SERPING1* in 9 probands diagnosed with HAE-1.

## Methods

### Subjects

Nine patients with HAE from different families were diagnosed with HAE-1 using clinical criteria (recurrent attacks of subcutaneous and/or submucosal edema) and laboratory criteria (decreased complement 4 (C4) levels and low antigenic level of C1-INH, with normal C1q). The clinical data of the patients are shown in Table 1. Healthy blood donors (n = 53) were recruited from a selected panel without an individual or familiar clinical history of angioedema and with normal C4 and C1-INH levels. A group of 9 patients with HAE-1 with missense mutations were utilized (Additional file 1: Table S1).

**Table 1:** Patients’ clinical and genetic characteristics

Proband number	Age	Gender	C1-INH antigenic levels (g/L)	CDS	Molecule effects	Exon	References
1	53	Female	0.03	c.44 delT	p.L15Rfs78 ×	Exon 2	
2	30	Female	0.09	c.289 C>T	p.Q97 ×	Exon 3	[19]
3	25	Female	0.04	c.296_303 delCCATCCAA	p.T99fs129 ×	Exon 3	[19]
4	30	Male	0.05	c.538 C>T	p.Q180 ×	Exon 3	
5	52	Female	0.04	c.786-787insT	p.N263Qfs296 ×	Exon 5	
6	46	Female	0.04	c.794 G>A	p.W265 ×	Exon 5	
7	50	Male	0.04	c.939 delT	p.H314Tfs320 ×	Exon 6	
8	42	Female	0.09	c.1214-1223 delCCAGCCAGGA	p.T405Ifs427 ×	Exon 7	
9	81	Male	0.08	c.1279 delC	p.L426fs428 ×	Exon 8	[19]

*C1-INH* C1 inhibitor, *CDS* coding sequence

### Genetic analysis

Genomic DNA was extracted from peripheral blood with the QIAamp DNA Blood Mini Kit (Qiagen, Hilden, Germany). DNA yield was calculated from the optical density (OD) at 260 nm and the purity by calculating the ratio of OD 260 nm and 280 nm by Multiskan GO with a cuvette (Thermo Fisher Scientific, Vantaa, Finland). Primers were designed for the analysis of the eight exons in the *SERPING1* gene according to a previous study [19]. The amplification reaction mixture (50 µl) for polymerase chain reaction (PCR) contained 5 µl of 10× *Pfu* DNA Polymerase Reaction Buffer with MgSO_4_ (Promega, WI, USA), 1 µl of dNTP mix, 1 µl each of sense/antisense primer, 2 µl of *Pfu* DNA Polymerase (Promega, WI, USA), 100 ng template DNA. The PCR program was performed on an Arkit thermal cycler 96-well block (Thermo Fisher Scientific, Vantaa, Finland). The amplicons were identified by electrophoresis on a 1.2% agarose gel and then sequenced at the Beijing Genomics Institution (Beijing, China). The mutations were identified by comparison to the reference sequence of the *SERPING1* gene X54486 (BA123456, GenBank).

Total RNA was isolated from peripheral blood by the QIAamp RNA Blood Mini Kit (Qiagen, Hilden, Germany), and its yield and purification were quantitated by OD 260 nm and 280 nm. The integrity of total RNA was analyzed by 1.2% agarose gel electrophoresis (see Additional file 2: Figure S1). cDNA was synthesized from 200 ng of total RNA by using the SuperScript^®^ III First-Strand Synthesis System (Invitrogen, CA, USA). Real-time PCR was performed on an ABI 7500 real-time PCR system (Applied Biosystems, CA, USA), and primer-specific amplification was performed in the presence of Power SYBR Green PCR Master Mix (Applied Biosystems, CA, USA). Glyceraldehyde-3-phosphate dehydrogenase (GAPDH) was used as the endogenous control to normalize the data.

### Measure of C1INH antigen and C4

The level of C1-INH antigen (C1-INHa) was measured by the chromogenic method on a BN™ II System (Dade Behring Marburg GmbH, Marburg, Germany). C4 concentration was measured by C4 Reagent, 2 × 100 Test Cartridge (Beckman Coulter, CA, USA). The normal ranges of C1-INH antigen and C4 used for reference were 0.21–0.39 g/L and 0.1–0.4 g/L, respectively.

### Statistical analysis

Statistical analyses were performed using SPSS 17.0 (version 17.0; SPSS Inc., Chicago, IL, USA). The Mann–Whitney test was used to compare the levels of mRNA between patients and controls and the levels of C1-INHa between patients with nonsense and patients with missense mutations. A *P*-value of < 0.05 was considered statistically significant.

### Ethics, consent and permissions

This study was approved by the ethics committee of our hospital. All participants gave their informed written consent for using their sample and publishing the results.

## Results

Nine probands from unrelated families were included in this study. According to the diagnosis criteria [1], all the probands were classified as HAE-1. The clinical characteristics for each patient are shown in Table 1.

### Mutations in the *SERPING1* gene

Nine mutations in the *SERPING1* gene were identified in nine probands with HAE-1: c.44delT, c.289C<T, c.296_303delCCATCCAA, c.538C<T, c.786_787insT, c.794G<A, c.939delT, c.1214_1223delCCAGCCAGGA, and c.1279delC (Table 1) which led to a premature stop codon in the mutation site or downstream of the mutation site. None of these mutations were present in healthy controls. Among them, c.289C<T, c.296_303delCCATCCAA and c.1279delC were reported previously [19].

### Expression of *SERPING1* mRNA

**Fig. 1:**
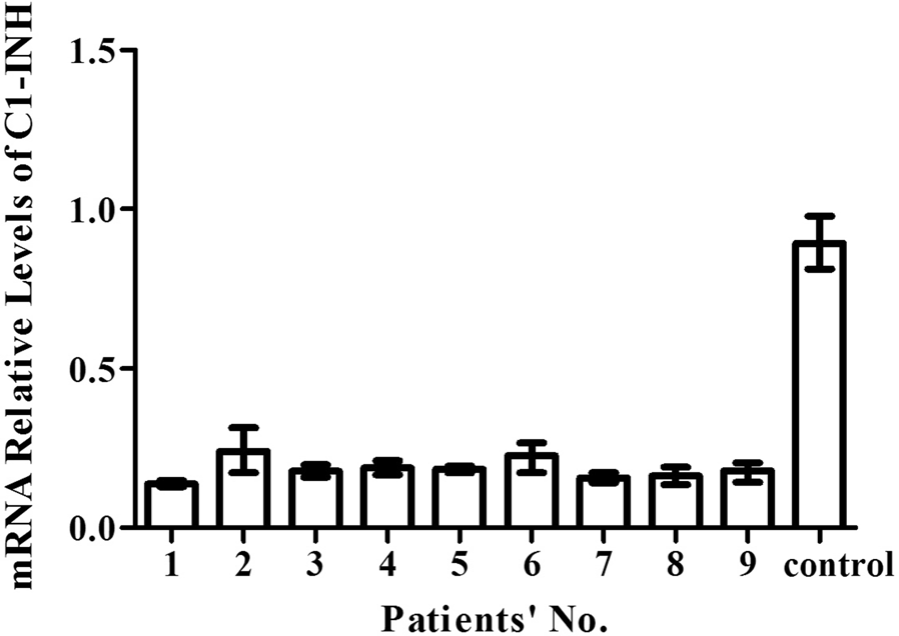
Expression of C1 inhibitor (C1-INH) mRNA. The mRNA levels in the studied HAE-1 patients were lower than those in healthy controls

The relative quantification of *SERPING1* mRNA was calculated by normalizing the ratio for *SERPING1* to GAPDH (shown in Fig. 1). The mean expression levels of *SERPING1* mRNA in the patients and the controls were 0.18 and 0.89, respectively. A significant reduction in *SERPING1* mRNA expression was found in patients compared to the controls (P < 0.001).

### Correlations of mutation types and clinical parameters

**Fig. 2:**
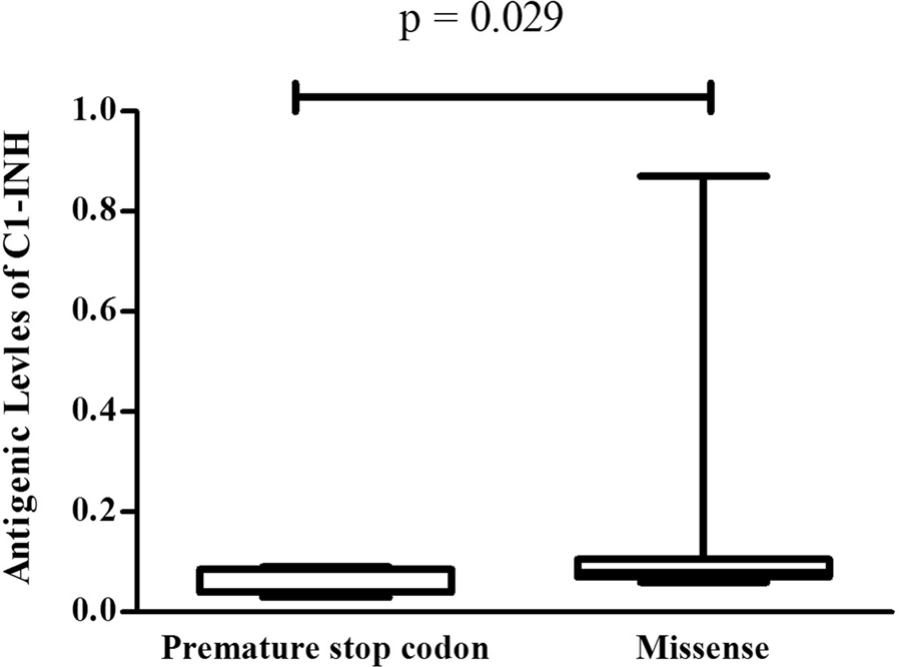
The levels of antigenic C1 inhibitor (C1-INHa) in patients with a premature stop codon and missense mutations. Premature stop codon mutations produced much lower levels of C1-INHa

The levels of C1-INHa were collected to serve as the comparison from 9 patients with missense mutations. The median (quartile 25, quartile 75) C1-INHa values in patients with premature stop codons and missense mutations were 0.04 (0.04, 0.08) g/L and 0.08 (0.08, 0.1) g/L, respectively. The difference in C1-INHa between the two mutation types was statistically significant (P < 0.05, Fig. 2).

## Discussion

Genetic defects in *SERPING1* were identified as the pathogenic factor for C1-INH-HAE. In 2011, 48 C1-INH-HAE patients diagnosed in our center were screened for *SERPING1* gene mutations, and a preliminary Chinese *SERPING1* gene mutation database was set up [19]. However, due to blood sample limitations, analysis of mRNA levels was not performed in that work. In the current study, 9 probands with HAE-1 from unrelated families were analyzed in terms of DNA sequence and mRNA expression. Nine mutations were identified as probably being responsible for the pathogenesis of HAE. c.289C<T, c.53C<T, and c.794G<A create premature stop codons at the 97th, 180th, and 265th amino acid sites of the sequence, respectively, which would lead to the termination of mRNA translation and the synthesis of truncated protein products. The crystal structure of C1-INH includes a reactive loop (RCL), the key structure of C1-INH responsible for interactions with target serine proteases [20]. These three kinds of termination deletions owing to nonsense mutations were located upstream of the RCL. Thus, synthesized proteins would lack the RCL and could not identify target proteases. As a result, these mutations would prevent their suppressive effect. For c.44delT, c.939delT, c.1214-1223delCCAGCCAGGA, c.1279delC, c.296-303delCCATCCAA and c.786-787insT, the number of deleted bases was not a multiple of three. Therefore, these deletions would alter the reading frame, form termination codons in the downstream sequence and result in the formation of premature stop codons at the 78th, 129th, 296th, 320th, 427th, and 428th amino acid sites of the sequence, which were located upstream of the reaction key, Arg^466^. Arg^466^ is a crucial residue of C1-INH that can bind to the active site of target proteases. Mutations impacting Arg^466^ would influence the recognition between C1-INH and its target proteases and result in dysfunction of C1-INH. Via nonsense-mediated mRNA decay [21], all the detected mutations would lead to synthesis defects in the protein, and the patients would present with low concentrations of C1-INH.

The expression of C1-INH mRNA was analyzed by real-time PCR. In comparison with the healthy control, the expression of C1-INH mRNA remarkably decreased in HAE patients and was only 20% of the normal level on average. This finding was in accordance with the results from several previous works [14–16]. However, a Korean study described normal expression of C1-INH mRNA in HAE-1 [17]. One possible reason for this finding might be that all the mutations analyzed in the Korean cohort were missense mutations or were located in the introns, whereas in the other previous works and this study, nonsense and frameshift mutations were taken into account. Premature termination codons formed by nonsense or frameshift trigger rapid mRNA degradation without being translated [21, 22], so mRNA expression is reduced. When considering the mutation types, a significant difference in mRNA levels was observed between healthy controls and patients with nonsense mutations but not in those with missense mutations [14]. It has been hypothesized that missense mutations might impact the posttranslation process [17].

In our previous work, nonsense and frameshift mutations in Arg^466^ appeared to cause lower C1-INHa expression than missense mutations [19]. Here, we confirmed the former observation. The C1-INHa expression level in 9 studied patients carrying premature stop codons was much lower than that in those with missense mutations.

There were certain limitations in our study. First, because the RNA was not obtained from patients carrying missense mutations, the correlation between mRNA expression and C1-INHa levels in different mutation types could not be estimated. Second, when comparing the level of C1-INHa in patients with nonsense and missense mutations, only 9 cases for each genotype were utilized, which might result in statistical bias or error. Thus, it is hard to draw a conclusion that a nonsense mutation in *SERPING1* could lead to more severe deficiency of C-INH.

## Conclusion

In summary, we investigated C1-INH gene sequences and mRNA expression in 9 probands with HAE-1. Nine different C1-INH gene mutations were identified, 6 of which were novel, expanding the mutation spectrum of C1-INH-HAE. In addition, compared with controls, C1-INH mRNA levels in patients carrying premature stop codons were remarkably reduced.

## Supplementary information

**Additional file 1: Table S1.** C1-INH antigenic levels of patients with missense mutation data.

**Additional file 2: Figure S1.** Total RNA electrophoresis on 1.2% agarose gel. 1-9: patients’ number.

## Data Availability

The datasets used during the current study are available from the corresponding author on reasonable request.

## Abbreviations

C1-INH: C1 inhibitor
C1-INHa: C1 inhibitor antigen
C4: Complement 4
GAPDH: Glyceraldehydes-3-phophate dehydrogenase
C1-NH-HAE: Hereditary angioedema with deficient or dysfunctional C1 inhibitor
HAE: Hereditary angioedema
HAE-nC1-INH: Hereditary angioedema with normal C1 inhibitor
OD: Optical density
PCR: Polymerase chain reaction
RCL: Reactive loop
