# Supplementary material for: Hereditary angioedema caused by a premature stop codon mutation in the SERPING1 gene
Source: Clin Transl Allergy. 2020 Nov 27;10:53. doi: 10.1186/s13601-020-00360-9 (PMC7694933; doi:10.1186/s13601-020-00360-9)
Supplement: Supplementary file 1 — Additional file 1: Table S1. C1-INH antigenic levels of patients with missense mutation data. [file 13601_2020_360_MOESM1_ESM.docx]

**Supplementary Table 1 C1-INH antigenic levels of patients with missense mutation data**

| **No.** | **C1-INH antigenic levels (g/L)** |
| --- | --- |
| l | 0.15 |
| 2 | 0.12 |
| 3 | 0.067 |
| 4 | 0.08 |
| 5 | 0.1 |
| 6 | 0.05 |
| 7 | 0.09 |
| 8 | 0.08 |
| 9 | 0.1 |
