# Supplementary figures and images for: Hereditary angioedema caused by a premature stop codon mutation in the SERPING1 gene
Source: Clin Transl Allergy. 2020 Nov 27;10:53. doi: 10.1186/s13601-020-00360-9 (PMC7694933; doi:10.1186/s13601-020-00360-9)

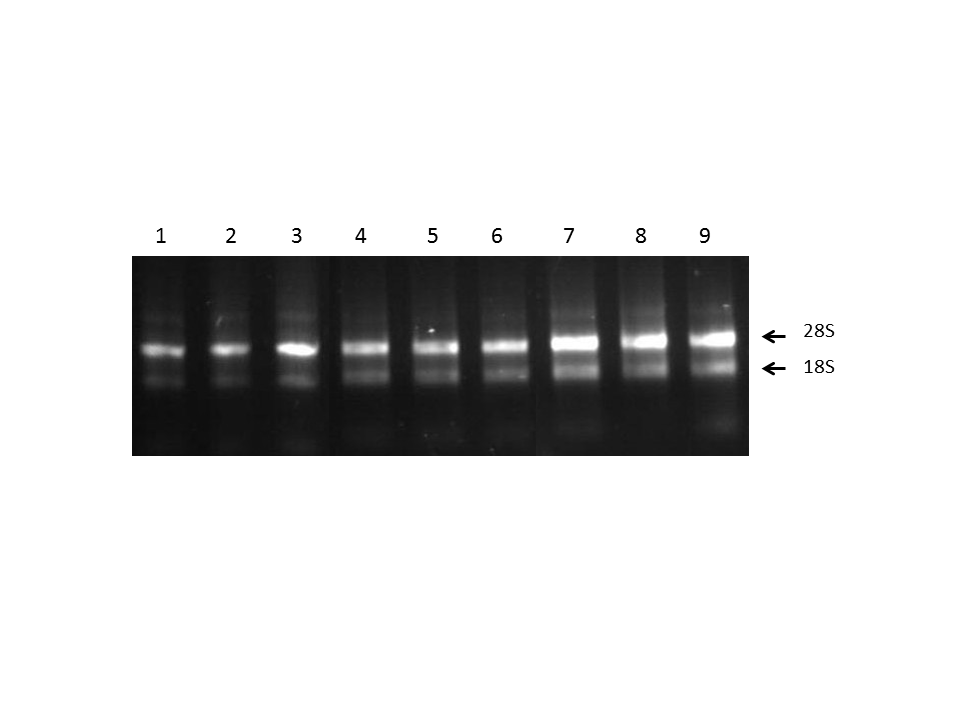

Supplement: Supplementary file 2 — Additional file 2: Figure S1. Total RNA electrophoresis on 1.2% agarose gel. 1-9: patients’ number. [file 13601_2020_360_MOESM2_ESM.tif]
